# Murine leukemia virus (MLV) P50 protein induces cell transformation *via* transcriptional regulatory function

**DOI:** 10.1186/s12977-023-00631-w

**Published:** 2023-09-12

**Authors:** Charbel Akkawi, Jerome Feuillard, Felipe Leon Diaz, Khalid Belkhir, Nelly Godefroy, Jean-Marie Peloponese, Marylene Mougel, Sebastien Laine

**Affiliations:** 1 Team R2D2: Retroviral RNA Dynamics and Delivery, IRIM, UMR9004, CNRS, University of Montpellier, Montpellier, France; 2 ISEM, CNRS, EPHE, Université Montpellier, IRD, Montpellier, France; 3 Team APIR: IRIM, UMR9004, CNRS, University of Montpellier, Montpellier, France

**Keywords:** Retrovirus, Murine leukemia virus, SD’, P50, Cancerogenesis, Leukemia, Transcription regulation, Transcriptomic

## Abstract

**Background:**

The murine leukemia virus (MLV) has been a powerful model of pathogenesis for the discovery of genes involved in cancer. Its splice donor (SD’)-associated retroelement (SDARE) is important for infectivity and tumorigenesis, but the mechanism remains poorly characterized. Here, we show for the first time that P50 protein, which is produced from SDARE, acts as an accessory protein that transregulates transcription and induces cell transformation.

**Results:**

By infecting cells with MLV particles containing SDARE transcript alone (lacking genomic RNA), we show that SDARE can spread to neighbouring cells as shown by the presence of P50 in infected cells. Furthermore, a role for P50 in cell transformation was demonstrated by CCK8, TUNEL and anchorage-independent growth assays. We identified the integrase domain of P50 as being responsible for transregulation of the MLV promoter using luciferase assay and RTqPCR with P50 deleted mutants. Transcriptomic analysis furthermore revealed that the expression of hundreds of cellular RNAs involved in cancerogenesis were deregulated in the presence of P50, suggesting that P50 induces carcinogenic processes *via* its transcriptional regulatory function.

**Conclusion:**

We propose a novel SDARE-mediated mode of propagation of the P50 accessory protein in surrounding cells. Moreover, due to its transforming properties, P50 expression could lead to a cellular and tissue microenvironment that is conducive to cancer development.

**Supplementary Information:**

The online version contains supplementary material available at 10.1186/s12977-023-00631-w.

## Introduction

All retroviruses share an elementary genomic organization with the *gag*, *pol* and *env* genes. They are divided into complex and simple families according to the complexity of their genomes. This complexity comes from the number of alternative splicing events of the retroviral genomic RNA, also called full-length RNA (FL RNA). In complex retroviruses, these splicing events (~ 30) lead to the synthesis of numerous regulatory and accessory proteins that are essential for the viral cycle, whereas simple retroviruses undergo only 1–2 splicing events. The links between retroviruses and cancer are now well documented. Both complex and simple retroviruses were found to be involved in cancer development. For complex retroviruses such as the Human T Leukemia virus 1 (HTLV-1), the viral protein Tax and HBZ proteins are crucial for cell transformation [1]. The cell transformation mechanisms are different for simple retroviruses. For instance, the Rous Sarcoma virus (RSV) includes v-Src, a viral-oncogene (v-onc) in its genome, which is essential for cancer development [2]. When simple retroviruses do not encode oncogene, the current model proposes that tumors arise following proviral integration near a cellular proto-oncogene, a phenomenon termed proviral insertional mutagenesis [3]. Viral integration deregulates the expression of cellular genes involved in cancerogenesis. The simple virus, Murine Leukemia Virus (MLV), induces tumors by insertion of its proviral DNA in regions near cellular oncogenes, thereby deregulating their expression [4, 5]. MLV insertional mutagenesis has been particularly valuable in deciphering molecular mechanisms of hematopoietic cancers, and for the identification of new cellular proto-oncogenes [6–10].

**Fig. 1:**
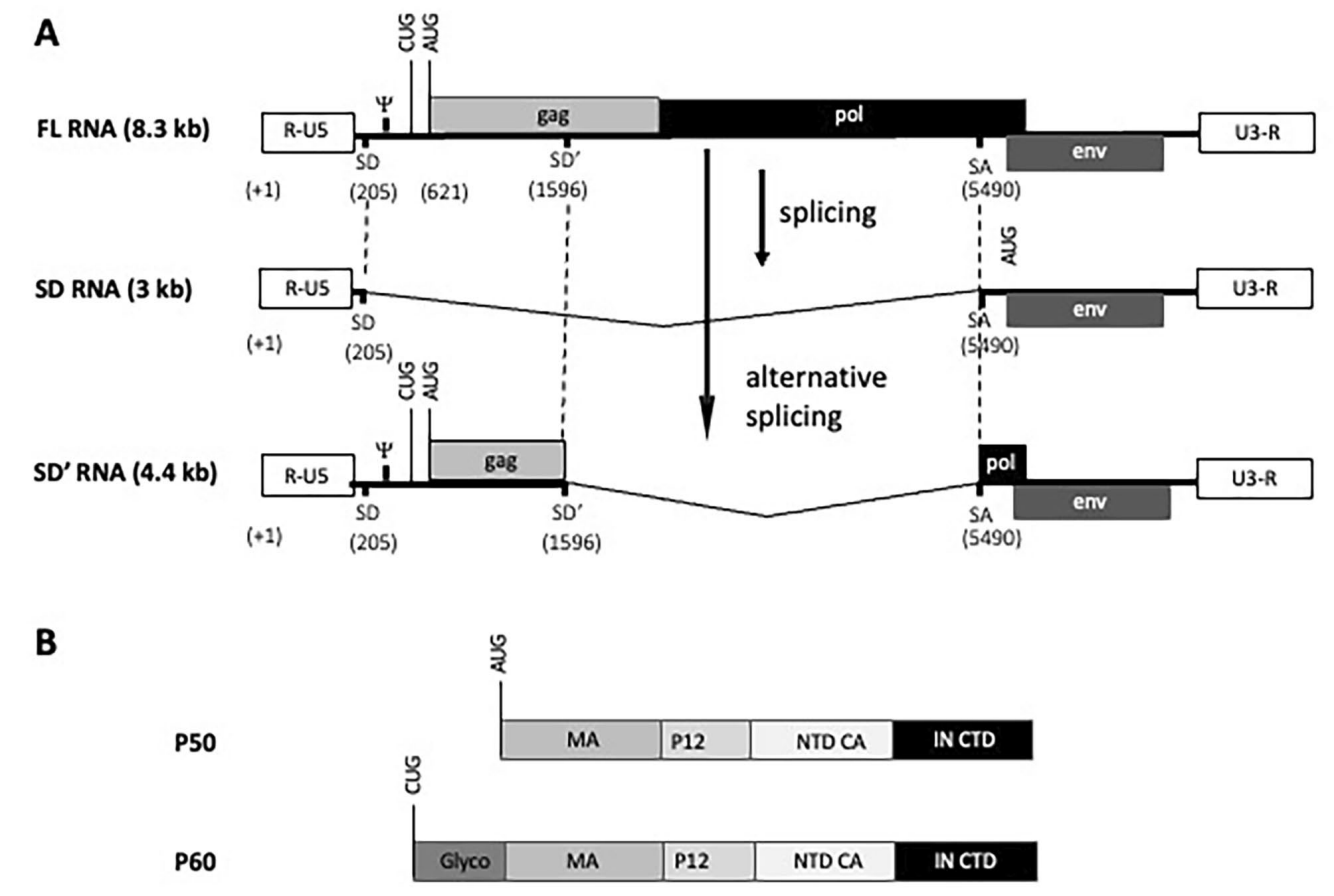
MLV RNAs and proteins encoded by SD’ RNA. (**A**) RNAs produced by MLV. Positions are numbered from cap site (+ 1) of the MLV gRNA. Non-coding elements R-U5 and U3-R are given at 5’ and 3’ ends, respectively, as well as *gag*, *pol* and *env* genes. Ψ depicts the packaging signal. Donor and acceptor splicing sites, SD, SD’ and SA, are indicated. The translation initiation sites of P60 and P50, CUG and AUG, respectively, are shown. (**B**) SD’ RNA translation. The different domains composing P50 and P60 are boxed. The N-terminal of CA and the C-terminal of IN are called NTD CA and CTD IN, respectively

Two strains of MLV, the simple prototypic retrovirus, are well-studied: Moloney (Mo-MLV) and Friend (F57). FL RNA of both MLVs encodes the structural Gag proteins and undergoes two splicing events (Fig. 1A): the canonically spliced SD mRNA encoding Env proteins and the alternatively spliced SD’ mRNA encoding the proteins P50 and P60. The subgenomic 4.4 kb SD’ mRNA is derived from a splice donor site (called SD’) within the MLV *gag* sequence and the canonical splice acceptor site (SA) located in *pol* gene [11] (Fig. 1A). SD’ was shown to be important for MLV biogenesis as an inactivating mutation of SD’ (without change in the *gag* amino acid sequence) reduces viral replication in murine cells [11]. We showed previously that SD’ RNA shares similar abilities with FL RNA throughout the viral life cycle. SD’ can be detected as a cDNA copy integrated into the host genome, demonstrating that that SD’ RNA is reverse transcribed and consequently behaves as a retroelement called SDARE for spliced donor-associated retroelement [12]. Moreover, SD’ RNA is efficiently packaged into progeny virions. A detailed analysis of the RNA content of MLV particles showed that SD’ RNA can form SD’/FL heterodimers [13, 14]. Since SD’/SD’ has been detected in vitro [13], we hypothesized that virions might contain only SD’/SD’ homodimers, without FL RNA. Thus, we tested whether particles containing SD’ RNA only could enter the cell and produce P50 protein. We have studied the role of P50 in this context as well as in MLV-infected cells.

Studies in vivo revealed that inoculation of a Mo-MLV mutant (MSD1/M1) harboring an inactive SD’ site in newborn Swiss mice changes the MLV leukemogenic properties [15]. Indeed, whereas WT MLV exclusively induces T lymphoma, the MSD1/M1 mutant can induce a broad panel of leukemia (T lymphoma, erythroleukemia, myelomonocytic leukemia and other leukemia with undetermined origin). The same mutant was analyzed in the model of MLV-induced myeloid leukemia (MML) in pristane-treated BALB/c mice [16]. In this model, SD’ is used for the production of an oncogenic gag-myb fusion RNA. However, we do not know whether the implication of SD’ splicing site in tumorigenesis might also involve SD’-encoded proteins (Fig. 1B). Indeed, SD’ mRNA encodes two proteins, P50 and P60, initiating at two distinct initiation codons in the same reading frame: AUG^gag^ and CUG^glyco-gag^, also used for the synthesis of Gag and Glyco-Gag polyproteins, respectively [17–20]. Like Glyco-Gag, P60 is expressed at lower level than P50 [12]. The P50 protein harbors the N-terminal Gag domain including the Matrix (MA), P12 and the first 110 amino acids of Capsid (CA) domain in frame with the last 116 amino acids of integrase (IN) domain from *pol* gene (Fig. 1B). P60 possesses the same domains with 88 extra amino acids in the N-terminal region. We showed that P50 fused to GFP localizes around the nucleus and mainly at the plasma membrane and is packaged in virions suggesting a role during early and late events of the MLV life-cycle [12]. In 2020, S. Ross’s team reported for the first time the role of P50 in counteracting the cellular restriction factor APOBEC3 in mice infected by MLV [21]. Like the HIV-1 auxiliary protein Vif, P50 overcomes APOBEC3 restriction by preventing its packaging into virions but without inducing mouse APOBEC3 degradation. APOBEC3 resistance was also ensured by Glycogag by limiting its access to the reverse transcription complex [22–24].

The perinuclear localization of P50 remains intriguing. P50 harbors the P12 and C-terminal domain (CTD) of IN that show chromatin association capacity [25–28]. P12 contains a nucleosomal-binding domain required for the association of MLV pre-integration complex to the host cell DNA during infection [27, 28]. The IN-CTD domain contains a BET interacting domain, which guides integration targeting by tethering the pre-integration complex near transcription start sites [29, 30]. All these elements suggest that P50 could also play a role in the nucleus. To this end, we have undertaken investigations into the putative role of P50 in carcinogenic processes and have discovered a transcriptional regulatory function of P50. Finally, we have performed a transcriptomic analysis to identify cellular genes whose expression is altered by the presence of P50.

## Results

### P50 can spread from cell to cell via viral particles harboring the SD’ retroelement

**Fig. 2:**
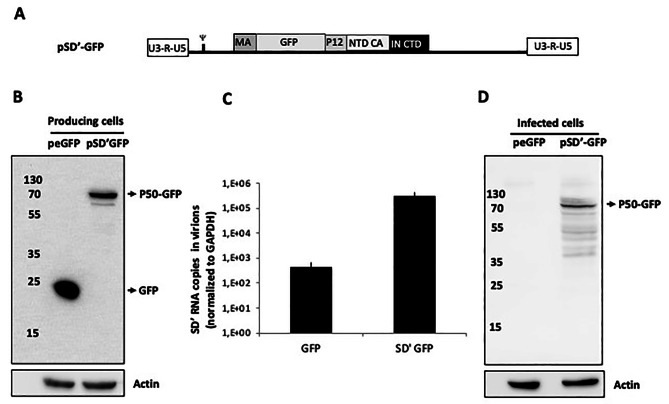
Infection with retrotransposon SD’. (**A**) Schematic representation of chimeric pSD’-GFP RNA. NIH 3T3 was co-transfected with a construct expressing GFP (peGFP) or P50-GFP (pSD’-GFP) and a molecular clone of MLV harboring a deletion of Psi (pMov Psi-). (**B**) Three days post transfection, cell extracts were analyzed by WB using anti-GFP and anti-actin antibodies. (**C**) Supernatants were collected and virus content was analyzed by RT-qPCR before being used to infect NIH 3T3 cells. (**D**) Three days post-infection cell extracts of infected cells were analyzed by WB using anti-GFP and anti-actin antibodies

SD’ RNA is selectively packaged in MLV virions. After virus entry, SD’ RNA is reverse transcribed and integrated into the host genome [12, 13]. However, SD’ expression, i.e. P50 production, in these infected cells has not yet been assessed. To do so, we co-transfected murine NIH 3T3 cells with plasmids expressing the SD’ retroelement or GFP vector as a control RNA that is not specifically encapsidated in virions, as well as a Mo-MLV molecular clone lacking its packaging signal (pMov9.1 Psi-) to produce virions containing only SD’ RNA as a genome [31]. Three days post-transfection, viral protein expression was analyzed by western blot (WB) and harvested culture media were used to infect NIH 3T3 cells. As GFP reporter gene was inserted in frame between the MA and the P12 domains in the P50 sequence (Fig. 2A), P50-GFP was detected as a 75 kDa protein by WB by using an anti-GFP antibody (Fig. 2B). Virions were purified from the supernatant and their content was analyzed by RT-qPCR showing the presence of encapsidated SD’ RNA (Fig. 2C). As previously reported [31], FL Psi- RNA was undetectable with values ≤ mock (4 × 10^1^ cps). Then, we infected NIH 3T3 cells with purified virions. After three days of infection, we detected the presence of P50-GFP (P75) in infected cells by WB (Fig. 2D, lane SD’GFP). P75 expression was not due to an unspecific packaging into MLV virion, since GFP was not found in infected cells (Fig. 2D, lane GFP). These results showed that particles harboring only SD’ RNA are infectious and the SD’ retroelement is transcribed and translated into the P50 protein.

### P50 induces cell proliferation and transformation

Having shown that P50 alone can be expressed, it is interesting to investigate its impact on the host cells. We previously showed that SD’ splice site is required for myeloid tumor development in vivo [16]. Nevertheless, nothing is known concerning the potential oncogenicity of P50. One of the hallmarks of oncogenes is their ability to enhance cell growth. To know if P50 is involved in cancer cell progression, we firstly asked whether expression of P50 alters proliferation of immortalized cells.

**Fig. 3:**
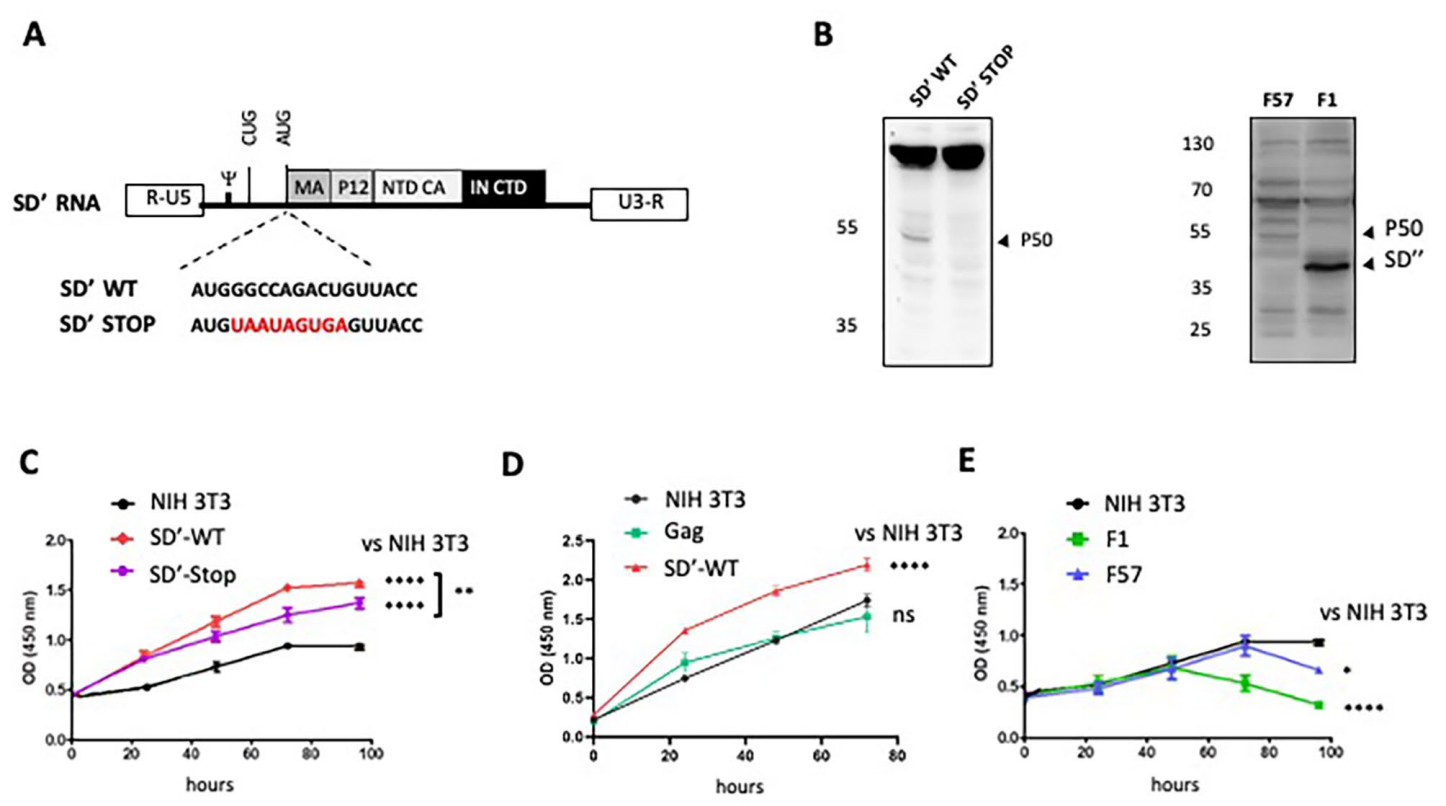
Effect of P50 on cell proliferation. (**A**) Schematic representation of SD’ RNA WT or with STOP mutation (in red) inhibiting the P50 expression. NIH 3T3 cells were stably transfected with either SD’ WT, SD’ STOP or Gag 2LTR or chronically infected with F57 or F1 viruses. (**B**) The expression of P50 was monitored by WB, using the anti-All-Friend antibody, in F57 and F1 infected cells and in SD’ WT- and SD’ STOP- stably expressing cells. SD” corresponds to the product (42KDa) of an cryptic splicing that is artificially woken up when SD’ alternative splicing was inactive [11]. Effect of expression of P50 or SD’RNA (**C**), MLV Gag (**D**) or F57 or F1 viruses (**E**) on cellular proliferation was monitored daily using CCK8-coloration assay (Dojindo) (CFM-cell free medium). Experiments were performed in triplicate and the significance of differences was assessed using a one-way ANOVA test (****p ≤ 0.0001 and *p ≤ 0.1, ns, not significant)

We stably transfected NIH 3T3 cells with a vector expressing a mutant SD’ RNA that no longer encodes P50 (Fig. 3A and B) [12]. In parallel, we also analyzed the role of P50 in infected cells. Therefore, we used NIH 3T3 cells chronically infected with either F57 or F1 mutant (not expressing P50) [11]. The absence or the presence of P50 was monitored by WB (Fig. 3B). As previously reported when SD’ splicing is inactive, an SD’’ protein is artificially produced with a larger NTD-CA that is no longer in phase with the IN sequence [11]. To monitor proliferation, cells were plated in a low-serum medium and analyzed daily for five days. There was a modest but significant effect of the SD’ RNA (Fig. 3C) and a very small additional increase of cell proliferation due to P50 protein.

As the impact of SD’ RNA could be due to the presence of SD’ site or the influence of LTR promoter after insertion in cellular genome, we tested the effect on cell proliferation of the Gag gene under the control of LTR promoters since it harbors the SD’ site and part of the P50 sequence except for the IN domain. This construct did not affect cell proliferation (Fig. 3D) thus confirming the importance of the full-length P50 protein in cell proliferation.

In the context of entire viruses, our results showed that F1 infected cells were less proliferative than those infected with the F57 virus (Fig. 3E). These results, compared to the proliferative effect observed when only P50 is expressed (Fig. 3C), showed that the absence of P50 inhibited the cell proliferation (Fig. 3E), although a role for SD’’ in inhibiting cell proliferation cannot be completely excluded. The decrease in proliferation observed in F57 infected cells was probably due to a cytopathic effect of the infection that occurs after 4 days post-infection. Taken together, these results showed that P50 increases cellular proliferation in cells. In order to know the role of P50 in MLV infection, all future experiments will be performed in the presence of P50 alone or in the viral context.

**Fig. 4:**
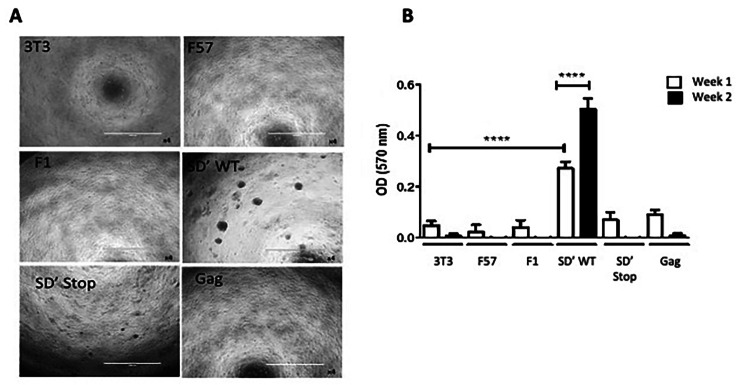
Effects of P50 on anchorage-independent growth. NIH 3T3 cells were stably transfected with SD’-WT, SD’-STOP or Gag or chronically infected with F57 or F1. Effect of P50 on anchorage-independent growth was monitored using soft agar assay. (**A**) Micrographs showing the colonies formed in the agar matrix at day 14. The scale bar corresponds to 1 mm (**B**) Absorbance-based quantitative analysis of the different cell lines. Experiments were performed in triplicates and error bars indicate SEM. The significance of differences was assessed using a one-way ANOVA test (****p ≤ 0.0001 and *p ≤ 0.1, ns, not significant)

To assess the possible impact of P50 on anchorage**-**independent growth, which defines transforming activity in immortalized fibroblasts [32], we monitored foci formation over a two-week period of NIH 3T3 cells infected with F57 or F1 and NIH 3T3 cells that stably expressed SD’-WT or SD’-STOP mRNA, by using a 96-well plate soft agar assay (Fig. 4). No foci appear in cells carrying empty vector, Gag-plasmid, or infected by F57 or F1. We observed foci with cells expressing SD’-WT (Fig. 4A and B). However, some little foci were observed with cells expressing SD’ STOP RNA but they failed to form living colonies (Fig. 4A and B), demonstrating that, contrary to P50, SD’ RNA induced cell proliferation but not cell transformation (Fig. 3C). Moreover, the lack of foci observed in F57, where P50 is expressed at a low level, suggests that the effect of P50 on anchorage-independent growth is dose-dependent.

**Fig. 5:**
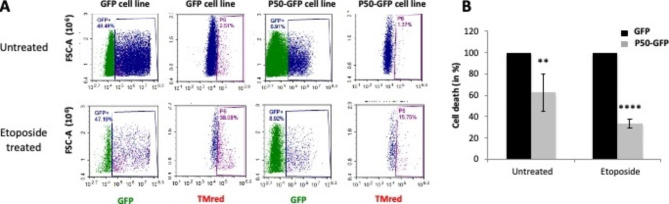
Effects of P50 on apoptosis mechanism. NIH 3T3 were transiently transfected with P50-GFP or GFP expressing plasmids and then treated or not with 25 µM of Etoposide. (**A**) After TUNEL treatment, cells were sorted by FACS. The areas circled in blue and violet represent GFP-positive cells and among them, cells undergoing apoptosis (TMred positive), respectively. A representative experiment is shown here. (**B**) The cellular mortality was analyzed in GFP or P50-GFP expressing cells. Cells undergoing apoptosis expressing GFP are used as reference (100%). The significance of differences was assessed using an unpaired Student’s *t*-test (****p ≤ 0.0001)

In parallel, to further investigate the mechanisms of cell transformation by P50, apoptotic activity was examined in NIH 3T3 stably expressing P50-GFP using TUNEL assay. The apoptotic level was measured by FACS analysis with cells treated or not by an apoptotic inducer. Cell expressing GFP were used as negative control. Briefly, cells were treated or not with etoposide (25 µM) for 24 h to induce apoptosis and the population of apoptotic cells was counted *via* the presence of Tm Red fluorescence revealing DNA breaking following apoptosis (Fig. 5A). Figure 5A showed that in the absence of apoptosis inducer, cells expressing P50-GFP markedly decreased the level of apoptosis (40 +/- 19%) compared to the GFP control. This effect is more pronounced in the presence of etoposide. Indeed, in this condition we observed a drastic decrease in cell mortality (65 +/- 5%). Our data (Fig. 5A and B) suggest that P50 protects the cells from apoptosis.

### P50 is partially localized in the nucleus

**Fig. 6:**
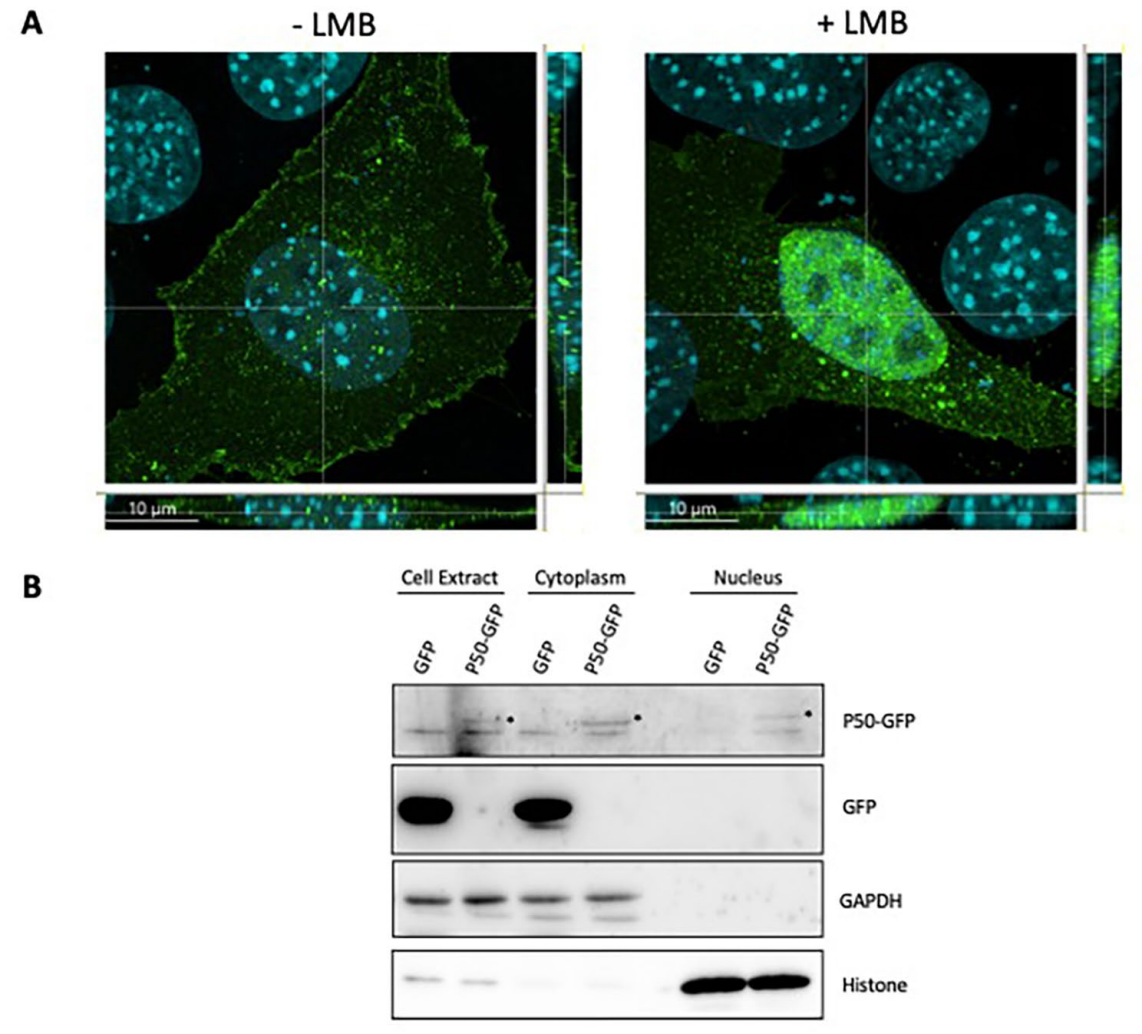
Subcellular localization of P50. (**A**) NIH 3T3 cells were transiently transfected with pSD’GFP (see Methods). Two-days post-transfection, cells were treated or not with 40 nM of LMB for 4 h and subcellular localization analysis was performed with confocal microscopy. Nuclei were stained with DAPI (blue) and P50-GFP appeared in green (GFP channel). Scale bar is 10 μm. (**B**) Total cell extracts, cytoplasmic and nuclear fractions of cells transfected with p57SD’/GFP or empty peGFP plasmid, were analyzed by WB using antibodies against GFP, GAPDH and Histone 3. The stars indicate the position of P50-GFP.

P50 implication in cell transformation could suggest a nuclear function of P50. Previous fluorescence microscopy analysis reported that P50-GFP is perinuclear and accumulates at the plasma membrane [12]. To further investigate P50 subcellular localization, we analyzed by using confocal microscopy the P50-GFP location in cells transiently transfected with pSD’-GFP under the control of MLV LTR promoter, treated or not with 40 nM of Leptomycin B (LMB), a CRM1 pathway inhibitor, as decribed in [33] (Fig. 6A). Without treatment, P50-GFP was mainly distributed in the cytoplasm and at the plasma membrane. Interestingly, a few dots of P50-GFP were also detected in the nucleus (Fig. 6A). Treatment with LMB significantly increases the amount of P50-GFP in the nucleus (Fig. 6A). Interestingly, this increase is characterized by a greater number of dots but also by greater overall fluorescence in the nucleus. This nuclear localization was further confirmed with cell fractionation experiments (Fig. 6B). The fractionation was controlled by the detection of the cytosolic protein GAPDH and the nuclear histone H3 protein. These controls confirmed that the weak signal of P50-GFP in the nuclear fraction was not due to a leakage from the cytoplasmic fraction.

### P50 is a transregulator of the MLV transcription

**Fig. 7:**
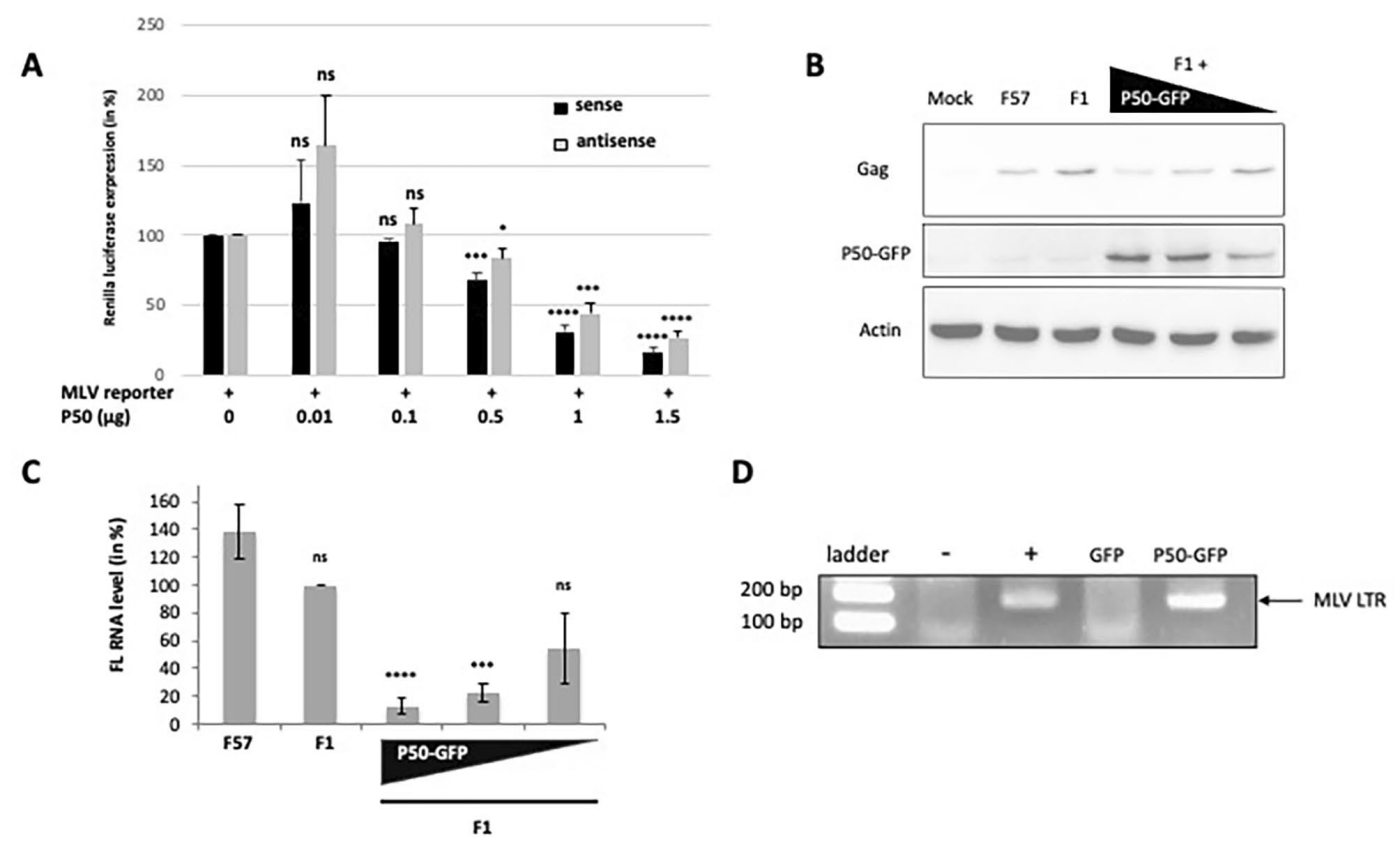
Trans-regulator activity of P50 on MLV promotor expression. (**A**) MLV promotor expression was monitored with luciferase assays. The NIH 3T3 cells were co-transfected with 1 µg of bidir MLV-LTR plasmid and increasing amounts of p57cDNASD’ construct (1.5 to 0.01 µg) expressing untagged P50. Luciferase activity was analyzed 48 h post-transfection and normalized to protein concentration, measured by the Bradford assay using BSA as a standard. The activity observed with the MLV reporter alone was used as a reference (100%). Experiments were performed in triplicates. Average luciferase activities (arbitrary units) for sense and antisense were 171,639 and 4122, respectively. Values were compared using unpaired Student’s t-test (ns: not significant, *p* ≤ 0.1 and ****p ≤ 0.0001). (**B**) NIH 3T3 cells were cotransfected with plasmids expressing the mutant F1-MLV and p50-GFP and 4 8 h post transfection, Gag or P50-GFP expressions were monitored in cells and supernatants by WB analysis and (**C**) cellular MLV FL RNA was quantitated by RT-qPCR. (**D**) Chromatin immunoprecipitation of P50. NIH 3T3 cells were co-transfected with plasmids expressing F1 and P50-GFP or GFP alone. Two days post transfection cell extracts were collected and used for a ChIP experiment with an anti-GFP antibody. DNA fragments co-precipitated with P50-GFP were analyzed by PCR using probes recognizing MLV promotor. The plasmid encoded MLV was used as PCR positive control (+) and cell extract from untransfected cells was used as PCR negative control (-)

The presence of P50 in the nucleus as well as the presence of a BET binding domain in CTD-IN domain and a nucleosomal-binding domain in P12 domain (Fig. 1B) suggest that P50 could regulate MLV transcription. Thus, we initially studied the impact of P50 on the transcriptional activity of the MLV LTR. Like HTLV-1, MLV harbors bidirectionnal activity of its promoter meaning that MLV RNA is transcribed from the 5’LTR and also to a lesser extent from the 3’ LTR [34, 35]. In order to monitor the effect of P50 on sense and antisense transcription, we used the reporter vector MLV-Luc bidir, expressing the Renilla luciferase under the control of MLV LTR promoter in the sense orientation and the Firefly luciferase in the antisense direction [34]. NIH 3T3 cells were cotransfected with MLV-Luc bidir, and increasing concentrations of p57cDNASD’ vector, expressing untagged P50. Two days after transfection, cells were collected and luciferase activity was measured. As shown in Fig. 7A, the lowest amounts of P50 did not significantly change the activity of MLV promoter in the sense direction (0.01 µg: 123 +/- 31% and 0.1 µg: 96 +/-1% of the control level without p57cDNASD’) while higher amounts (1 and 1.5 µg) significantly reduced luciferase activity (69 +/-5% and 84 +/-4% reduction, respectively). While the effect on the antisense transcription was less marked, it was nevertheless significant (1.5 µg: 73 +/-5% reduction).

To study the P50 effect on MLV expression, NIH 3T3 cells were co-transfected with a F1-MLV and a vector expressing P50-GFP in dose-dependent manner. Total proteins were extracted and separated by SDS-PAGE to monitor the expression of Gag translation by WB (Fig. 7B). Interestingly, results showed a dose-dependent decrease of Gag translation along with an increase of P50-GFP level, which corroborates the results obtained with the luciferase assays (Fig. 7A). Furthermore, we monitored the levels of FL RNA by RT-qPCR (Fig. 7C). This quantification revealed a strong decrease of FL RNA (87 +/- 6%) in the presence of high level of P50-GFP and a decrease of 45 +/- 11% was still observed at a low level of P50 (Fig. 7C). All together these results strongly suggested that P50 inhibits the transcription of the MLV promoter.

To further analyze the P50 ability to interact with MLV LTR *in cellulo*, chromatin immunoprecipitation (ChIP) assays were performed in NIH 3T3 cells co-expressing F1-MLV and P50-GFP or GFP alone as negative control. Briefly, 48 h post transfection, formaldehyde crosslinking was performed and DNA fragments interacting with P50-GFP or GFP proteins were isolated with magnetic beads coated with GFP antibodies. Bound DNA was identified by PCR using specific probes targeting LTR promoter (Fig. 7D). A band corresponding to the expected size on LTR promoter was obtained with P50-GFP (lane 4), and not with the GFP control (lane 3). This result suggests that P50 allows co-immunoprecipitation of the LTR promoter. However, the specificity for the LTR is not exclusive since the P12 domain can interact with nucleosomes and therefore the entire MLV genome might be also pulled down.

### IN-CTD domain contributes to transciption regulation ability of P50

**Fig. 8:**
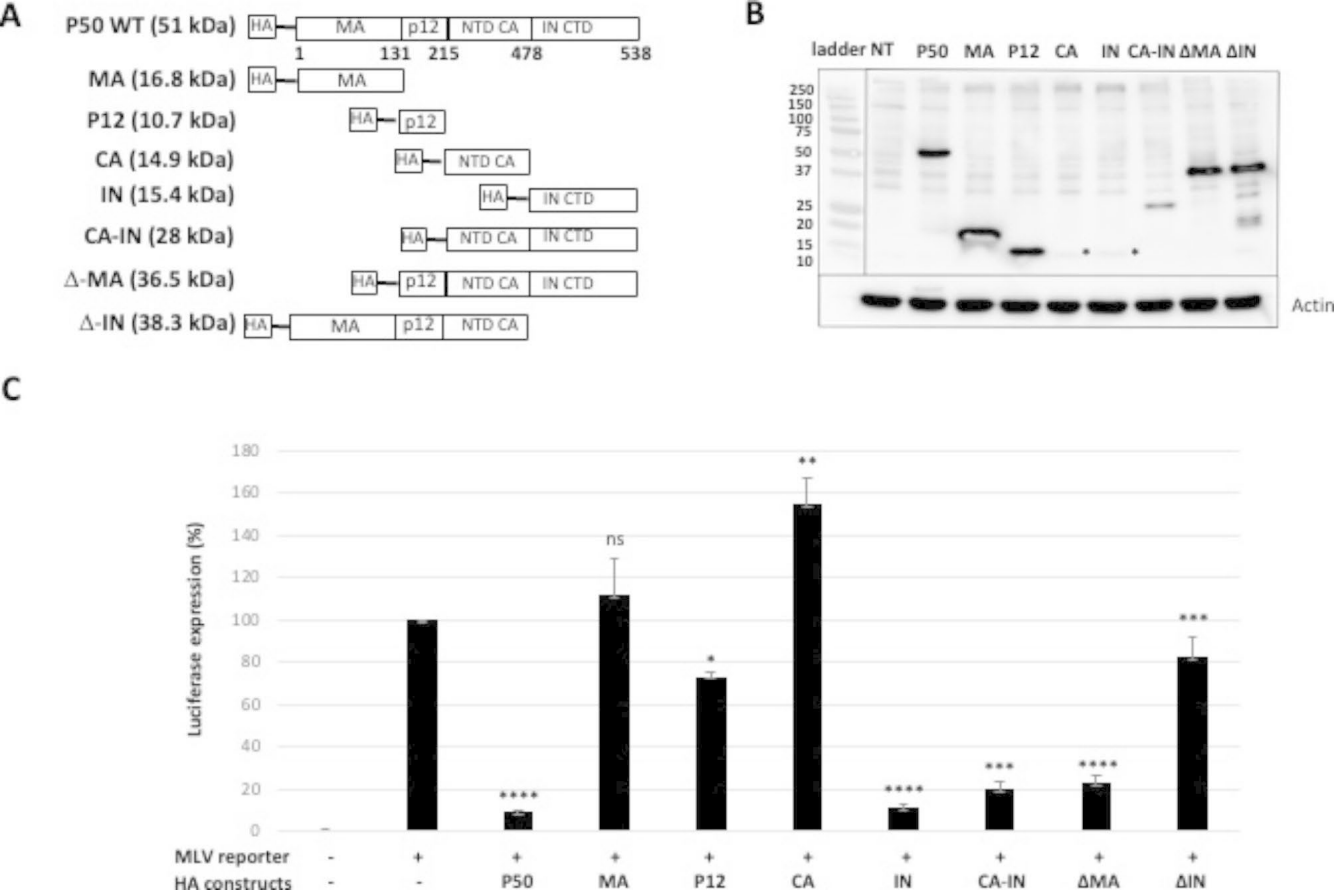
Effects of P50 domains on MLV promotor activity. (**A**) Schematic of the different HA-P50 constructs. The amino acid positions of P50 domains are given in bold. The molecular weights are in brackets. (**B**) Expression of the different HA-P50 constructs was checked by WB analysis with anti-HA antibody. The stars show the localization of N-term CA and C-term IN domain. Experiments were performed in triplicates. (**C**) MLV promotor expression. NIH 3T3 co-transfected with bidir MLV-LTR and pRK5-HA-P50 or empty pRK5-HA constructs. Luciferase activity was analyzed 48 h post-transfection and values obtained with only bidir MLV-LTR plasmid was referred as 100%. Experiments were performed in triplicates and the significance of differences was assessed using an unpaired Student’s *t*-test (ns: non significative, ****P ≤ 0.0001, ***P ≤ 0.001, **P ≤ 0.01 and *P ≤ 0.1)

P50 shares domains with Gag or Pol proteins (Fig. 1). Interestingly, in addition to the MA and P12 domains, P50 harbors the chimeric CA-IN domain formed by an in-frame fusion of the N-terminus of CA with the C-terminus of IN. In the presence of MLV protease, the complete maturation of P50 results in the chimeric CA-IN protein. We performed luciferase assays to identify which domain of P50 contributes to the regulation of LTR transcription. Multiple combinations of domains were constructed and tagged with HA in N-term (Fig. 8A). Expression of these truncated constructs were monitored by WB analysis. All mutants were well expressed except the CA and the IN domains which were weakly expressed (Fig. 8B). These constructs were co-transfected with the MLV-luc reporter in NIH 3T3 cells and Renilla luciferase activity was quantified (Fig. 8C). The results showed that P12 domain slightly decreased the luciferase activity (27 +/- 2%) whereas MA domain had no effect. Unexpectedly, the CA domain increased the luciferase activity (55 +/- 12%). Interestingly, the IN domain despite its low expression level, showed similar transrepression activity (89 +/- 2%) to that observed with entire P50 protein (Fig. 8C). Thus, IN domain acts as the functional domain of P50 that represses MLV LTR expression. Conversely, deleted mutants were constructed to test the role of IN in transcriptional repression. The results confirmed the role of IN in MLV LTR regulation. Indeed, the ∆IN lead to the highest luciferase activity (87 +/-6%) among the deletion mutants. However, the ∆IN did not reach the transcription level of the reporter alone suggesting that another domain could also contribute to the transregulation of P50. The P12 domain is probably the best candidate as it slightly decreased luciferase activity (Fig. 8C).

### IN domain of P50 is involved in the transforming capacity of P50 

**Fig. 9:**
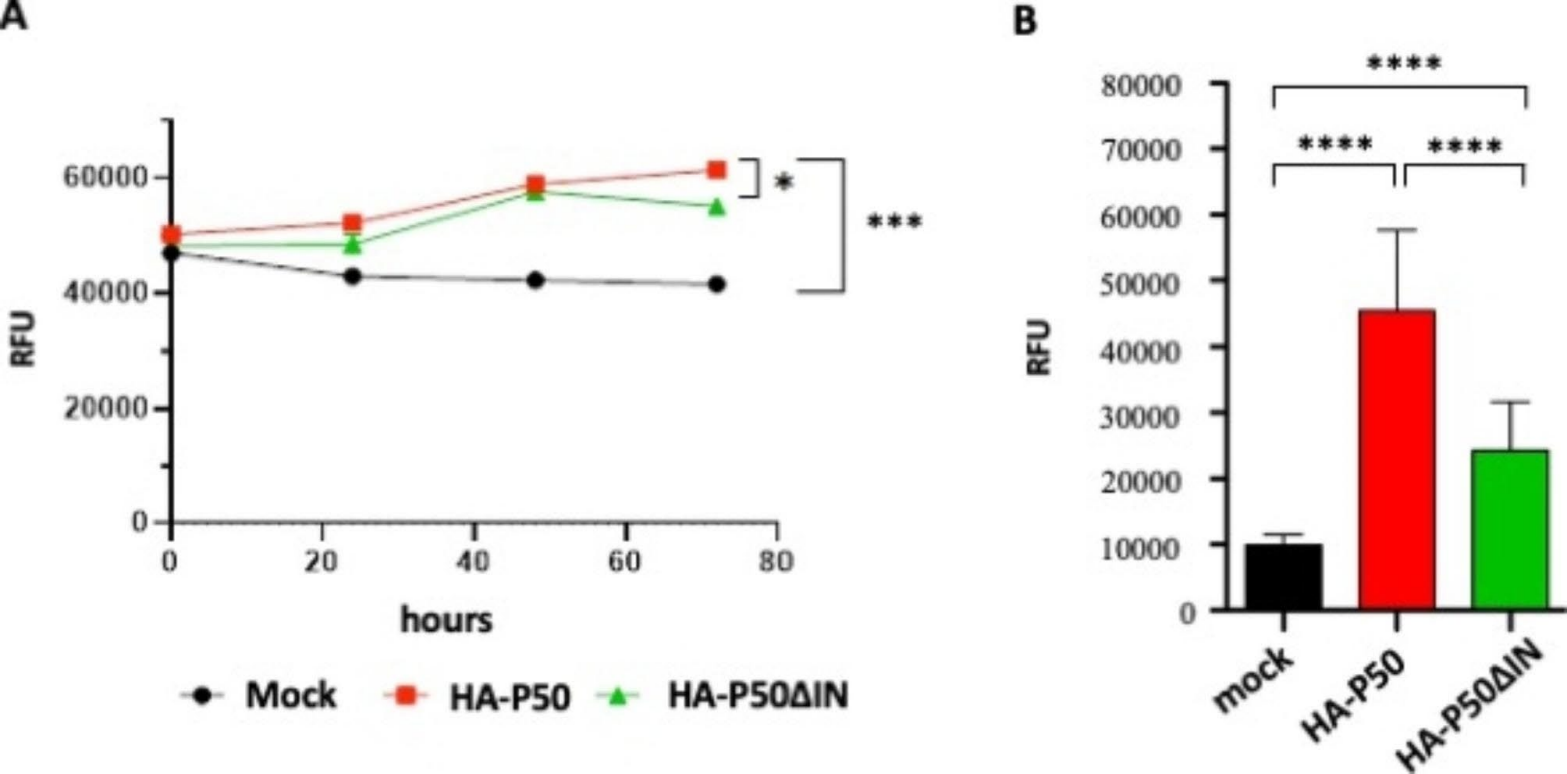
Effect of IN domain on cell proliferation and anchorage-independent growth. NIH 3T3 transfected by pRK5-HA-P50 or pRK5-HA-P50∆IN and then stably expressing HA-P50 or HA-P50∆IN were performed. (**A**) Cell proliferation was monitored daily using Presto Blue-coloration assay (Invitrogen) (CFM-cell free medium). (**B**) Effect of HA-P50 and HA-P50∆IN on anchorage-independent growth was monitored using soft agar assay. Absorbance-based quantitative analysis of the different cell lines grown in the agar matrix at day 14 was performed with Presto Blue-coloration assay (Invitrogen). Results are given in Relative Fluorescence Units (RFU). Experiments were performed in triplicates and error bars indicate SEM. The significance of differences was assessed using a one-way ANOVA test (****p ≤ 0.0001 and *p ≤ 0.1, ns, not significant)

To further investigate the role of the IN domain in P50-induced cell transformation, we performed cell proliferation and growth experiments on soft-agar with NIH 3T3 cell lines stably expressing HA-P50∆IN (Fig. 9). To monitor proliferation, cells were plated in a low-serum medium and analyzed daily for three days. The results showed that, unlike HA-P50, the presence of HA-P50∆IN had less impact on cell proliferation (Fig. 9A). In parallel we also tested the impact of HA-P50-∆IN on anchorage-independent growth. As previously described, we monitored the cell viability over a two-week period of NIH 3T3 cells stably expressing HA-P50 and HA-P50∆IN mRNA, using a 96-well plate soft agar assay (Fig. 9B). Interestingly, we observed that HA-P50∆IN had less impact on anchorage-independent growth. Taken together these results suggest that the effect of P50 on cell proliferation and anchorage-independent growth is dependent on its IN domain.

### P50 regulates transcription of cellular genes

**Fig. 10:**
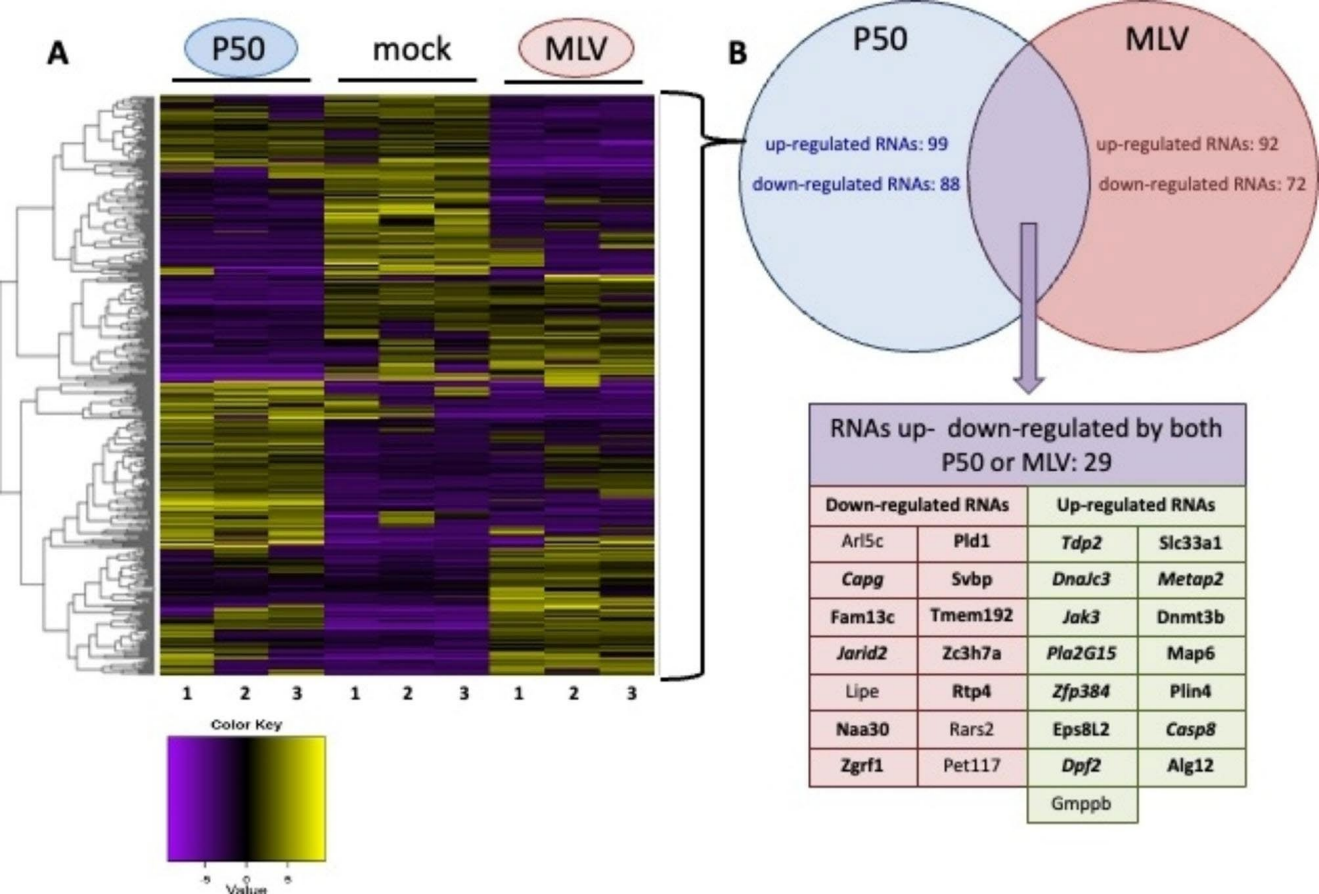
Transcriptomic analysis. (**A**) RNA populations from mock NIH 3T3, cells chronically infected by F57 (MLV) and cell line stably expressing P50 (P50) were analyzed in transcriptomic assays (in triplicate: 1, 2 and 3). The dendrogram representing the result of the clustering of all pairwise dissimilarities between the transcript expressions is represented on the left. The (**B**) RNAs regulated by P50 alone (blue circle) or in viral context (red circle) are represented and RNAs identically regulated in both conditions are represented in violet and listed in the table. RNAs from genes implied in cancer are in bold and those particularly involved in leukemia in bold Italic

Next, we wondered if the P50 transcriptional regulation activity could be extended to cellular genes. For this purpose, a comparative transcriptomic study was performed with NIH 3T3 (mock), NIH 3T3 chronically infected with F57 and NIH 3T3 stably expressing P50 (Fig. 10). A total of 83,173 transcripts showed a measured level of expression under the different conditions. The comparison between MLV infected cells and P50 stably expressing cells to identify differentially expressed genes, using DESeq2 under iDEP with FDR < 0.001 and a fold change > 8, identified 165 upregulated and 185 downregulated transcripts (list on request). Figure 10 shows the expression profile of the regulated genes in all conditions. In infected cells (MLV), we found 92 up-regulated RNA and 72 down-regulated RNAs. The same proportion of regulated RNAs are found up-regulated and down-regulated, 99 and 88 respectively, in P50 stably expressing cells (P50) [see additional file 1 and 2 respectively]. Among these genes, 29 gene expression profiles observed in MLV and in P50 varied in a similar way. Interestingly, most of these genes are linked with cancer processes (Pld1, Fam13c, Svbp, TMem192, Zc3h7a, RTP4, Naa30, Zgrf1, Slc33a1, Dnmt3b, Map6, Plin4, Eps8L2, Alg12, Capg and Tdp2) [36–52]. Moreover, we found that some genes are also important for leukemia development (Jarid2, Metap2, Jak3, Pla2G15, Zfp384, Casp8 and Dpf2) [53–59].

The transcriptomic analysis revealed that P50 modulated some genes involved in cancer biogenesis which is in good correlation with the effects of P50 on cell proliferation and transformation processes (Figs. 3, 4 and 5). Taken together, these results revealed that P50 is involved in cancerogenesis by acting as a transcription regulator.

## Discussion

As previously reported, the SD’ RNA acts as a splice donor-associated retroelement (SDARE). Indeed, it is selectively packaged in infectious particles and after cell entry, it is reverse transcribed by the viral RT and integrated as proviral SD’ by the IN [12]. Packaging is not a particularity of FL RNA and viral spliced RNAs also dimerize (for review, [60]) and are specifically packaged in virions [61]. The SD’ RNA can dimerize [13] and since it carries the Psi packaging signal, it is specifically packaged with FL RNA in infectious particles. Our team reported the existence of MLV particles harboring heterodimers FL/SD’ RNA suggesting that homodimers SD’/SD’ can also be packaged [14]. Here, we produced virions containing only SD’ RNA (and no FL RNA) that are still infectious since P50 is expressed in infected cells. Therefore, P50 might spread to cells neighboring the primary infected cell. In this context, P50 is produced after transcription of the proviral SD’ and not from SD’ mRNA originated from FL RNA splicing, suggesting that SD’ retroelement can spread among cells. The presence of a retroviral protein in neighboring cells has already been reported for HIV-1 secreted Tat protein [62]. This regulatory protein is able to enter cells and disturb several cellular pathways by modifying the expression of cellular genes [63]. No such protein was reported yet for simple viruses, such as MLV. Here, we revealed for the first time that MLV uses another strategy based on the release of infectious particles harboring the retrotransposon SDARE encoding the regulatory protein P50. To our knowledge, such a mechanism has never been described in the literature.

SD’ RNA and/or P50 protein contribute to MLV infectivity (F57 and Mo-MLV) and pathogenesis [11]. Although a role for P50 has recently been reported in counteracting the restriction factor APOBEC3 [21], P50’s role during infection remains poorly understood. Here, we showed that P50 was also involved in transcription regulation and cell transformation. The hallmarks of cancer were divided in ten biological capabilities: proliferative signaling, evading growth suppressor, resisting cell death, enabling replicative immortality, inducing angiogenesis, activating invasion and metastasis deregulating cellular energetics, avoiding immune destruction, tumor-promoting inflammation and genome instability and mutation [64]. We demonstrated that P50 ticks three of these hallmarks: proliferative signaling (Fig. 3), evading growth repressor (Fig. 4) and resisting cell death (Fig. 5), indicating that P50 harbors oncogenic properties. More specifically, we have shown that the effects of P50 on certain hallmarks of cancer (proliferative signaling and evading growth repressor) are due to its IN domain (Fig. 9). Nevertheless, the molecular mechanisms of oncogenesis remain to be fully elucidated. In agreement with this ability, a low amount the P50-GFP was found to be localized in the nucleus and its transport across the nuclear envelope is CRM1 pathway dependent (Fig. 6A). Like the regulatory proteins Tat of HIV and Tax of HTLV [65, 66], which transactivate viral transcription (for review, [67, 68]), P50 also acted as a regulatory protein. Indeed, P50 transrepressed the viral transcription and consequently decreased the FL RNA translation (Fig. 7). This regulatory function was mediated by its IN-CTD domain with a lesser contribution from its P12 domain (Fig. 8). IN domain interacts with the cellular BET proteins that guides MLV integrations near transcription start sites [30, 69]. The involvement of the BET proteins in the activity of P50 on cellular or viral promoters will need to be further investigated. Interestingly HIV-1 IN-CTD domain contributes to viral transcription regulation by Tat [70] *via* interactions with TAR region [71]. P12 domain, on the other hand, harbors a nucleosomal-binding domain facilitating MLV integration [27, 72]. It is possible that the P12 domain of P50 acts in synergy with IN-CTD domain to modulate the transcription regulation activity of P50.

Until now there was no evidence of a role of P50 in oncogenic processes. Our transcriptomic study revealed for the first time that in addition to acting on MLV promoter, P50 deregulated mRNAs of several cellular genes, including genes involved in essential carcinogenesis processes (Fig. 10). Interestingly, among the RNAs regulated by MLV or P50 alone, we found the mRNAs of the genes Ldlr, Tnr, Mapre2, Fnbp1, Hmgcr, Rara and Camk2d, which have been previously identified as the MLV insertion sites in different cell types [7, 9, 10, 39]. Due to these transregulatory capacities, P50 could interact directly or indirectly with transcription factors or cell promoters. This phenomenon has been reported for HTLV-1 whose onco-protein Tax affects viral RNA transcription as well as cellular RNAs leading to cell transformation [73]. Among cellular genes which are transregulated by P50, the majority encode proteins involved in cell-cycle regulation, or proteins found deregulated in cancers and leukemias. Thus, we found that the expression of several mRNAs encoding proteins involved in cell proliferation (Jak3, Fam13c, Pla2g15, Arl5c, Naa30, Pld1, Tmem192) is affected by the expression of P50 protein alone and in the viral context [37, 38, 40, 43, 55, 56, 74]. This ability to affect cell growth is found in other viral oncoproteins. Indeed, the NS3 and NS5A proteins of the hepatitis C virus promote the proliferation of hepatic cells via the β-catenin pathway [75]. Interestingly, we showed P50 altered transcription of RNAs encoding several proteins such as Fam13c and Jak3, which are transcriptional activators of the β-catenin gene that promotes cell growth [76, 77]. Therefore, it is quite conceivable that P50 protein increases cell proliferation *via* the β-catenin pathway.

Similarly, several mRNAs encoding proteins involved in growth inhibitory signals (Jarid2, Metap2) were affected by P50 [53, 78]. The dysregulation of Jarid2 gene is observed during Kaposi sarcoma-associated herpesvirus infection and leads to cell transformation [79]. Metap2, on the other hand, is differentially expressed in cells derived from mice developing an acute murine leukemia [54]. Thus, all these data suggest that P50 could act on several signaling pathways leading to insensitivity to growth inhibitory signals.

Regarding genes controlling cell apoptosis, we detected variation in the expression of several mRNAs controlling cell death (Naa30, Tmem192, Casp8) [40, 43, 58]. As a large fraction of P50 is in the cytosol, protection from apoptosis could also be due to the interaction of P50 with cellular partners. Such inhibition of apoptosis is observed for hepatitis viruses (for review, [80]) and further investigations will be necessary to test this possibility.

**Fig. 11:**
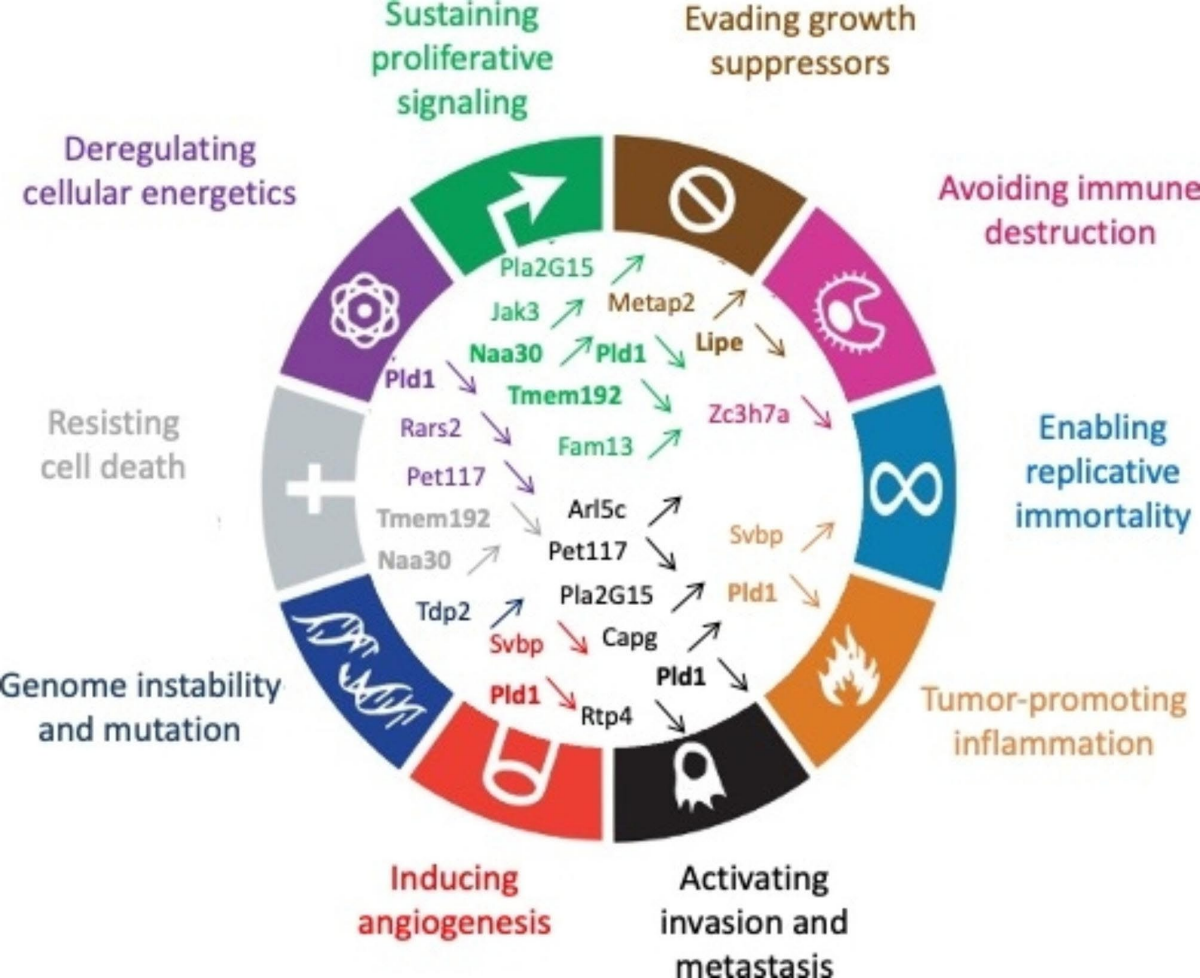
P50 and cancerogenesis. Schematic of classification according to the hallmarks of cancer of different proteins encoded by altered cellular mRNAs in the presence of P50 alone and in viral context (adapted from [64]). The mRNAs encoding proteins involved in different deregulated pathways are in bold. Deregulated genes playing a role in a distinct hallmark of cancer are in same color. Upregulation and downregulation of genes are noted by upward and downward arrows, respectively

Our results shed light on the pathways used by P50 to regulate mechanisms involved in loss of contact inhibition, immortalization or proliferation. However, other mechanisms of carcinogenesis are also impacted by P50 (Fig. 11), since several variations in expression of mRNAs controlling cellular energy metabolism were detected: Pet117, which belongs to the prostaglandin G/H synthase pathway [81, 82], Lipe that is involved in the regulation of lipogenesis [83], or Rars2 involved in the biogenesis of the phosphorylating oxidative chain [84, 85]. Genes involved in other characteristics of cancers, such as inflammation, genomic instability or escape from the immune system were also impacted by P50. We thus detected an increase in mRNAs encoding Pld and Svbp proteins, responsible for angiogenesis and inflammation [86, 87], Tdp2 involved in genomic instability, and Zc3h7a involved in escape from the immune system [51, 88]. The transcriptomic study also revealed altered expression of several aforementioned genes encoding proteins involved in different distinct deregulatory circuits such as Pld, Tmem192, Naa30 and Lipe.

Overall, our results showed that P50 affected the expression of cellular genes involved in cellular transformation and cancerogenesis. SD’ retrotransposon and the encoded P50 protein promote viral replication that could result in a cellular and tissue microenvironment conducive to the development of cancers [89, 90]. In contrast to HIV-1 Tat protein, which is secreted from infected cells and then enter into uninfected cells [91], P50 is disseminated *via* the SD’ RNA contained in infectious viral particles. To our knowledge, this kind of dissemination is unique in the retroviral family.

## Methods

### Plasmid constructions

Plasmids were constructed by using common cloning techniques and propagated in the E. coli DH5α at 37°c or STBL2 strain at 30 °C for plasmids carrying LTR to prevent any recombination. Finally, all constructs were sequenced.

Viral production was based on co-expression of Mo-MLV molecular clone deleted of its Psi signal (pMov9.1 Psi-) [31] with peGFP-N2 (Clontech) as control or pBSKEco SD’GFP (pSD’GFP) vector. pSD’GFP was generated by fragment exchange using XhoI and HindIII digestion sites between pBSKEco-p12-GFP-MS2 WT [92] and pSD’ [12] respectively.

For proliferation and transformation experiments, F57 strain and Friend F1 SD’ mutated molecular clone (F1) are described in [11]. The p57cDNASD′ vector (SD’ WT), expressing SD′ RNA and proteins, and the p57cDNASD′ stop (SD’ Stop) vector which expressing SD′ RNA only were previously described [12]. The p2LTR-Gag-F57 vector (Gag 2 L), expressing Gag under LTR promotor was obtained by deleting a 4424 nucleotides fragment in pF57 molecular clone with SacII and EcoRI enzymes. The obtained cohesive strand ends were filled with klenow enzyme and religated with T4 DNA ligase. The plasmids expressing HA-P50 (pRK5-HA-P50) and HA-P50∆IN (pRK5-HA-P50∆IN) were constructed as explained below.

TUNEL analysis was performed with plasmid expressing P50-GFP (p1LTR-P50-GFP). p1LTR-P50-GFP was generated by fragment exchange using AflIII and HindIII digestion sites between p57cDNASD’ and p57SD’/GFP described in [12]. The plasmid thus created was digested with AatII then treated with Klenow fragment and religated in order to introduce a STOP codon upstream of the ATG encoding P50.

Finally, the transactivating experiments were performed with Luciferase assays and ChIP analyses. Luciferase assays were carried out with MLV reporter vector (pAsLuc(Fire)-MLV-Luc(Reni)) containing Renilla and Firefly luciferase under LTR promotor which was generously provided by JM Mesnard [34]. Plasmids expressing P50 (p57cDNASD’), P50 tagged with HA or the multiple combinations of domains of P50 tagged with HA were obtained by amplification with standard PCR of P50 or every domain (alone or in combination) and inserted in pRK5-HA (addgene®) using EcoRI digestion. For ChIP analyses, the Mo-MLV molecular clone (pMov) [31] was used as positive control and the plasmid p1LTR-P50-GFP expressing P50-GFP (quoted above) was used for the experiment. Details of plasmid constructions will be provided on request.

### Cell culture and transfection

NIH 3T3, NIH 3T3 Packaging cell line [11] and NIH 3T3 chronically infected by F57 or F1 strains were grown in DMEM medium with penicillin-streptomycin (100 U/mL) and 10% foetal bovine serum (FBS) at 37°C and 5% CO2. Several stable NIH cell-lines were established by co-transfecting them with p57cDNASD’, p57cDNASD’stop, p2LTR-Gag-F57, pRK5-HA-P50 and pRK5-HA-P50∆IN plasmids and in a ratio of 10/1 the pGkNeo plasmid carrying the neomycin resistance. We used JetPei method according to the manufacturer’s instructions (Polyplus Transfection) and selection was started two days after transfection by adding G418 drug (100 mg/mL). After 1-month, all clones were trypsinized and mixed in a new dish.

### Western blotting

Protein analysis was performed by western blotting. Cells were scraped and lysed with the Complete lysis-M kit (Sigma) supplemented with protease inhibitor cocktail according to the manufacturer’s instructions. Total protein concentration was determined by Bradford assay using a BSA standard set (Fermentas). 100 µg of cell lysate Proteins was loaded on 12% SDS-PAGE and were electro-transferred onto nitrocellulose membrane. Gag was detected using a rat anti-capsid (CA) antibody (1/500, hybridoma H187, from B. Chesebro and M. Miyazawa). Actin was detected with an anti-actin (1/500, Sigma). P50 was detected with a Goat anti-All-Friend antibody (1/2000, from M. Sitbon). HA epitope was detected with a rabbit anti-HA antibody (1/4000, Abcam). After incubation with a peroxidase-conjugated (HRP) secondary antibody, ECL fluorescence was recorded by a CCD chemiluminescence camera system (ChemiDoc™ MP Imaging System, Bio-Rad).

### RNA analysis by RT-qPCR

RNA was extracted from cells with TriReagent (MRC) according to the manufacturer’s instructions and was quantified by RT-qPCR as described in [93]. After extraction, RNA samples were treated with RQ1 DNase (Promega) in the presence of RNasin (Promega) for 25 min at 37 °C, followed by phenol–chloroform and chloroform steps and finally precipitated with ethanol. After 70% ethanol wash, RNA pellets were dissolved in RNAse free water. RNAs were quantified by measuring optical absorption at 260 nm. For RT-qPCR, the RT step was performed with Expand reverse transcriptase (Roche) and initiated with an oligo (dT) primer or the same antisense primer used for the qPCR reaction. 2.5 µL of the RT reaction were engaged in qPCR reactions [93]. The following primers were used for FL or Psi + RNA: sFM76 5ʹ-GTGGTCTCGCTGTTCCTTGGGA sense and aM275 5ʹ-GCAGGCGCATAAAATCAGTCATAG antisense, for Psi- RNA: sMLV5356 CCCTGTACCGAGCCCGCAACAC sense and aFM5620 GCGGACCCACACTGTGTC antisense and for GAPDH: sGAPDH721 5ʹ-GCTCACTGGCATGGCCTTCCGTGT sense and aGAPDH921 5ʹ-TGGAAGAGTGGGAGTTGCTGTTGA antisense.

### Virus production and cellular infection

A total of 1.6 × 10^6^ NIH 3T3 cells were co-transfected with pMov9.1 Psi- and pSD’GFP expressing P50-GFP or empty peGFP-N2 plasmid as control in 10 cm-plates with JetPei method according to the manufacturer’s instructions (Polyplus Transfection). Two days post transfection, cells were collected and analyzed by Western blot, and the virus-containing media (10 ml) were collected, centrifuged 5 min at 1500xg and filtrated through a 0,45 µM filter. The supernatant was then ultracentrifuged on a 20% sucrose/phosphate-buffered saline (PBS) cushion at 30,000 rpm for 1h30 at 4 °C. The pellet was resuspended in 400 µL of complete DMEM. Viruses were then added to 3 × 10^5^ NIH 3T3 seeded the day before in a 6-wells plate in the presence of polybrene (10 µg/mL). Two hours post infection, 2 mL of complete DMEM were added. 24 h after infection, cells were trypsinised and transfer in a new 10 cm-plate. Three days post infection, infected cells were collected and proteins were analyzed by Western blot using anti-GFP antibody and normalized with anti-actin antibody.

### Cell fractionation

NIH 3T3 cells were seeded and transfected with p57SD’/GFP or empty peGFP plasmid as described above. 48 h after transfection, 2.6 × 10^5^ cells were harvested and subcellular fractionation was conducted with PARIS™ Kit with method according to the manufacturer’s instructions (Invitrogen, Carlsbad, CA, USA). In parallel, 2.6 × 10^5^ cells were scraped and lysed with the Complete lysis-M kit (Sigma) as described above to obtain cell extract. Half of nuclear or cytoplasmic fraction and cell extract was loaded on PAGE gel for Western blot analysis. Mouse anti-GFP (1/2000, Roche) was used as primary antibody to detect GFP and P50GFP. Cytoplasmic fractions were checked with anti-GAPDH (1/2000, Ambion) and nuclear fraction with rabbit anti-H3 (1/2000, Abcam) antibody.

### Cell proliferation

Cell proliferation was determined using cell counting kit 8 (CCK8; Dojindo or PrestoBlue; Invitrogen) according to the manufacturer’s protocol. Briefly, 5 × 10^4^ of each stable cell lines were plated in 96-well plates in DMEM medium supplemented with 1% heat-inactivated fetal bovine serum (FBS). 10 µl of substract solution were added daily to each well, followed by incubation for 4 h. The cell viability in each well was determined by reading the optical density at 450 nm for CCK8 or by measuring the fluorescence intensity (RFU) with PrestoBlue at 560/590 nm on a TECAN SPARK 10 M (TECAN).

### Anchorage-independent growth (soft agar cell culture) in 96-well plates

Soft Agar cell culture in 96-well plates was performed according to [94]. A total of 10^5^ stably transfected NIH 3T3 cells/well were seeded in 96-well plates with a bottom layer of 0.6% Select agar-DMEM (Invitrogen) and a top layer of 0.3% Select agar-DMEM. Fresh DMEM with 2% FCS was added to the top layer of the soft agar twice a week. The cells were allowed to grow in the humidified 37 °C incubator with 5% CO2 for 1–2 weeks. Colony growth and cell viability were measured using Prestoblue (Invitrogen -A13261) by reading the OD at 570 nm or the fluorescence intensity (RFU) at 560/590 nm on a TECAN SPARK 10 M (TECAN).

### Microscope analysis of P50-GFP

A total of 2.3 × 10^5^ NIH 3T3 cells were grown on a gelatin-coated coverslip in a 6-well plate. 2 µg of pSD’GFP were transfected as described above and 48 h after transfection, cells were treated or not with 40 nM of LMB (LC Laboratories) for 4 h as described in [33]. Then, cells were fixed with Formaldehyde (3.7%) for 10 min at RT. Then, Coverslips were washed twice with PBS and then mounted with vectashield + DAPI (1/2000, Thermo Fisher Scientific). Cells were imaged on a confocal laser scanning microscope LSM980 (Zeiss) equipped with a 63x/1.4 NA oil objective and 0.2 μm z-stacking space, with a minimum of 20 stacks per cell at Montpellier Rio Imaging (MRI) facilities. Images were deconvolved with Huygens Professional (version 22.10) and, maximum intensity projections and orthogonal views were prepared in Imaris Bitplane (version 10.0).

### TUNEL analysis

NIH 3T3 cells were transfected with p1LTR-P50-GFP or peGFP-N2 in a 6-well plate as described above, 24 h post-transfection, ± 25 µM of Etoposide drug (SIGMA) was added and followed by incubation for 24 h. Cells were collected and fixed with 3.7% formaldehyde for 10 min at RT. Then cells were washed twice with PBS and permeabilized with 0,05% Triton X-100/PBS for 10 min at RT. Cells were treated with TUNEL® kit (TMR red-Roche) according to the manufacturer’s protocol. Cells were then treated for FACS analysis using NOVOCYTE ACEA by exciting with 488 and 561 nm lasers at Montpellier Rio Imaging (MRI) facilities.

### Transregulation experiments

For luciferase assays, 2.3 × 10^5^ NIH 3T3 cells were seeded and transfected 24 h later, using JetPei method as described above, with 1 µg of the pAsLuc(Fire)-MLV-Luc(Reni) and different concentrations of p57cDNASD′ (1.5 to 0.01 µg) or 1 µg of HA-P50 and derivate constructs. 48 h post-transfection, cells were washed with cold PBS and then lysed in 1x passive lysis buffer (Promega), as described by the manufacturer. Luciferase assays were performed in a centro XS3 LB 960 microplate luminometer (Berthold Technologies) with respectively the Genofax A and Genofax C kit (Yelen). Luciferase activities were normalized with concentration of cell extract, determined by Bradford assay using a BSA standard set (Fermentas). For analysis of P50 effect on MLV promoter, NIH 3T3 were transfected, as described above, with F1/P50-GFP ratios of 1/2, 1/1 and 1/0.5. 48 h post-transfection, Gag and p50-GFP expressions were determined by Western blot analysis and the MLV FL RNA level was analyzed by RT-qPCR.

### Chromatin immunoprecipitation assay

A total of 1.6 × 10^6^ NIH 3T3 cells were co-transfected with 10 µg of plasmid expressing p1LTR-P50-GFP and 10 µg of F1 molecular clone using JetPEI in a 10 cm plate as described above. 48 h post-transfection, proteins from 10^7^ cells were cross-linked to DNA with 3.7% formaldehyde for 10 min at RT. Cells were washed twice with PBS and harvested by scraping. Nuclei were isolated by incubation of cross-linked cells with 1 ml of Cell Fractionation Buffer Kit PARIS™ (Invitrogen) for 5 min on ice and then pelleted by centrifugation (500xg). The nuclei were washed with 500 µl of Cell Fractionation Buffer Kit PARIS™ (Invitrogen) and incubated with 500 µL of Nucleus Lysis Buffer for 3 h on rotator at 4°C. The chromatin was fragmented by adding 5 µl of MNase enzyme (SimpleChip®) at 37°C for 20 min to an average size of 200 to 500 bp. Cell debris were pelleted by centrifugation (20,000×g, 4°C), and supernatants were collected. Chromatin was diluted in 50 mM Tris-HCl, 167 mM NaCl, 1 mM EDTA, 0.01% SDS, 1.1% Triton X-100, protease inhibitor mix. To reduce nonspecific background, antibodies were preincubated with 15 µl Dynabeads and protein G (DYNAL®) according to the manufacturer’s instructions. The antibody-bead complex was then added to chromatin samples, followed by incubation overnight at 4°C. Beads were washed once with low-salt buffer (2 mM Tris-HCl, 150 mM NaCl, 0.1% SDS, 1% Triton X-100, 2 mM EDTA), once with high-salt buffer (20 mM Tris-HCl, 500 mM NaCl, 0.1% SDS, 1% Triton X-100, 2 mM EDTA), once with LiCl wash buffer (10 mM Tris-HCl, 0.25 M LiCl, 1% Nonidet P-40, 1% sodium deoxycholate, 1 mM EDTA), and once with Tris-EDTA buffer. Chromatin was eluted from the beads in elution buffer (100 mM NaHCO3, 1% SDS) for 20 min at room temperature. DNA was incubated 5 h at 65°C and proteins were eliminated by a proteinase K digestion (1 h at 45°C). DNA was purified using the NucleoSpin® Gel and PCR clean-up kit and analyzed by PCR using One Taq® DNA Polymerase. To target the MLV LTR, the respective forward and reverse primers used were MLV sFM76: 5’GTGGTCTCGCTGTTCCTTGGGA3’ and MLV aM275 (5’GCAGGCGCATAAAATCAGTCATAG 3). As positive control we performed the PCR on molecular clone pMov9.1 and as a negative control, the PCR was performed on purified DNA from non-transfected cell extract.

### Transcriptomic analysis

#### mRNA isolation and sequencing

For each condition, three biological replicates were collected. Total RNA was extracted from cells with TriReagent (MRC) according to the manufacturer’s instructions. RNA samples were treated with RQ1 DNase (Promega) in the presence of RNasin (Promega) for 25 min at 37 °C. RNA was extracted with phenol–chloroform then chloroform and finally precipitated with 100% ethanol. RNA pellets were washed with 70% ethanol and dissolved in water. Libraries of each replicate were constructed and sequencing (100 bp, paired-end) was performed using an Illumina HiSeq2000 sequencer by the Montpellier MGX sequencing platform (http://www.mgx.cnrs.fr/).

#### Differential expression analysis

Most bioinformatics analyses were performed by the MBB platform in Montpellier (http://mbb.univ-montp2.fr/). For each condition (CNT, CCI and CES) 3 biological replicates were sequenced to provide a mean of 50 million paired-end reads per sample. Pre-processing of the raw RNA-seq data to remove contaminants, adapters, low-quality sequences and unpaired reads was performed with TrimGalore (https://github.com/FelixKrueger/TrimGalore) with standard parameters (quality 30 and length 50 bp). The New Tuxedo protocol [95] was used for the assembly of transcripts and the quantification of gene expression levels before analyzing differential expression with DESeq2. First, reads from each sample were mapped to the mouse reference genome (GRCm38) with HISAT2-2.1.0 [96] with default parameters and –dta to provide *alignments tailored for transcript assemblers.* To assemble transcripts following this protocol, StringTie-1.3.3b was provided with Mus_musculus.GRCm38.90.gtf as a reference annotation gene model file downloaded from ensembl web site (ftp://ftp.ensembl.org/pub/release-75/gtf/mus_musculus/). This guided assembly was provided a parameter to limit the processing of read alignments to only the assembled transcripts matching the reference transcripts. The StringTie’s acompagning script (http://www.ccb.jhu.edu/software/stringtie/dl/prepDE.py) was then used. The dendrogram representing the result of the clustering of all pairwise dissimilarities between the transcript expressions was obtained with the help of the Trinity tool (https://github.com/trinityrnaseq/trinityrnaseq/wiki/Trinity-Differential-Expression).

### Electronic supplementary material

Below is the link to the electronic supplementary material.

**Additional file 1:** Table S1. Cellular RNAs up-regulated by the P50 MLV protein.

**Additional file 2:** Table S2. Cellular RNAs down-regulated by the P50 MLV protein.

## Data Availability

All data generated or analyzed during this study are included in this published article and its Additional files 1 and 2.
